# Background Characteristics of Basic Health Examination Participants: the JPHC Study Baseline Survey

**DOI:** 10.2188/jea.13.216

**Published:** 2007-11-30

**Authors:** Motoki Iwasaki, Tetsuya Otani, Seiichiro Yamamoto, Manami Inoue, Tomoyuki Hanaoka, Tomotaka Sobue, Shoichiro Tsugane

**Affiliations:** 1 Epidemiology and Biostatistics Division, National Cancer Center Research Institute East.; 2 Cancer Information and Epidemiology Division, National Cancer Center Research Institute.

**Keywords:** basic health examination, participants, selection bias, population-based study

## Abstract

Background: Although epidemiologic studies including the Japan Public Health Center-based Prospective Study on Cancer and Cardiovascular Disease (JPHC study) have frequently used the basic health examination participants as study subjects, their background characteristics have rarely been investigated. The aim of this study is to clarify the background characteristics of participants and to discuss their impact on epidemiologic studies.

Methods: Subjects were 43,140 (Cohort I) and 34,892 (Cohort II) respondents aged 40-59 years who completed a self-administered questionnaire in 1990 or 1993-94 by the JPHC Study. Respondents whose data of the basic health examination were also available were defined as participants. We compared their sociodemographic factors, personal medical history, and lifestyle-related factors with those of non-participants.

Results: Participants tended to be older and less educated. They were more likely to engage in agriculture, forestry and fisheries or to be self-employed persons, or homemakers. Male participants smoked less and were more likely to drink alcohol beverage moderately. Female participants smoked and drank less but tended to participate more in sports and physical exercise in their leisure time. Both male and female participants tended to eat fruits and green vegetables more often than non-participants. In short, participants had a different socioeconomic status from non-participants and a favorable lifestyle profile, especially among women. These findings were principally consistent between the two cohorts.

Conclusion: These differences between participants and non-participants in the basic health examination might cause a selection bias that limits the application of the results to only participants in the basic health examination.

Many epidemiologic studies in Japan have targeted participants in the basic health examination (*Kihon-kenkou-shinsa*) under the Health and Medical Service Law for the Aged (*Roujin-hoken-hou*) because of the ease in obtaining their consent to participation and the possibility of high response rates.^1^^,^^2^ This could lead to a selection bias and limitation of external validity. Although the selection bias has been assessed in epidemiologic studies based on a questionnaire survey that showed respondents had better health status, higher education and income, and healthier lifestyle than non-respondents,^3^^-^^8^ few have dealt with the selection bias of participants in the basic health examination.

In a large population-based prospective study in Japan, the Japan Public Health Center-based Prospective Study on Cancer and Cardiovascular Disease (JPHC study),^9^^-^^12^ we collected not only information from a self-administered questionnaire but also health examination data and blood samples that were provided by only participants in the basic health examination. It is necessary to clarify the background characteristics of participants in the basic health examination before we analyze the data obtained only from such participants because this could help us to consider the possible selection bias and external validity of the findings. This issue also arises in connection with other epidemiologic studies that targeted participants in the basic health examination.^1^^,^^2^ In examining the background characteristics of participants, we divided respondents to the baseline questionnaire into two groups, participants in the basic health examination and non-participants, then compared sociodemographic factors, personal medical history, and lifestyle-related factors between groups.

## METHODS

### Study cohort

This study was conducted as a part of the JPHC study, which began in 1990 for cohort I, and involved five Public Health Centers (PHC): Ninohe PHC in Iwate Prefecture, Yokote PHC in Akita Prefecture, Saku PHC in Nagano Prefecture, Ishikawa PHC in Okinawa Prefecture, and Katsushika PHC in the Tokyo Metropolitan area. Cohort II of the study, begun in 1993, involved six other PHCs: Kasama PHC in Ibaraki Prefecture, Kashiwazaki PHC in Niigata Prefecture, Tosayamada PHC in Kochi Prefecture, Arikawa PHC in Nagasaki Prefecture, Miyako PHC in Okinawa Prefecture, and Suita PHC in Osaka Prefecture.^9^^-^^12^ The five PHC areas in Cohort I were selected based on the variation in mortality rate for stomach cancer from our previous ecological study, in which randomly selected subjects were intensively examined.^13^^-^^15^ The six PHC areas in Cohort II were selected considering geographical distribution and feasibility. The study population was defined to be all residents aged 40-59 years old in Cohort I and 40-69 years old in Cohort II at baseline who registered their address in 27 administrative districts (city, town, or village) supervised by nine PHCs except for Katsushika and Suita PHCs. The present study includes 54,498 residents (27,062 men and 27,436 women) in Cohort I and 62,398 residents (30,651 men and 31,747 women) in Cohort II after excluding subjects in Katushika and Suita PHCs due to different definitions of study population. As a baseline survey, we conducted a self-administered questionnaire among all study subjects, but collected health examination data and blood samples only from participants in the basic health examinations. Therefore, available data of the baseline survey differed for each subject. However, all subjects in the JPHC Study Cohort have been followed for their mortality, migration, and incidence of cancer and cardiovascular disease irrespective of whether or not they responded or provided health examination data or blood samples. A detailed design of the study has been reported elsewhere.^16^ This study was approved by the institutional review board of the National Cancer Center, Japan. Cohort subjects have been informed of the study design and results by annual or biannual newsletters, while the general population has had access to relevant information through our website (http://www.epidemiology.jp/).

### Baseline survey

**Figure 1.**
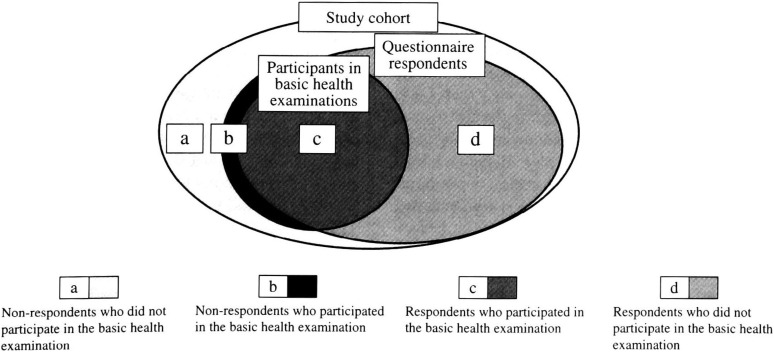
The relationship between study cohort and each baseline survey

In this study, we used questionnaire and health examination data from the baseline survey. Figure 1 shows the relationship between a study cohort and each baseline survey by Venn diagram.

A self-administered questionnaire was distributed to all cohort subjects in 1990 (Cohort I) and 1993-94 (Cohort II), mostly by hand and partly by mail. The questionnaire consisted of items covering sociodemographic characteristics, personal medical history, smoking status, habitual intake of foods and alcoholic beverages, physical activity and other lifestyle-related factors. The questionnaire used for Cohort II was somewhat modified. A total of 43,149 (79%) (20,665 (76%) men and 22,484 (82%) women) in Cohort I and 52,256 (84%) (24,804 (81%) men and 27,452 (86%) women) in Cohort II subjects returned their questionnaire (Figure 1, (c+d)/(a+b+c+d)). Telephone interviews were used to obtain complete answers when information was missing.

We collected the health examination data from the local municipalities involved with their permission. They provided all data obtained from persons aged 40-59 years in the Cohort I area and aged 40-69 years in the Cohort II area who underwent the basic health examination under the Health and Medical Service Law for the Aged within each survey year (Cohort I in 1990, Cohort II in 1993-94). We conducted record linkage between the health examination data and our master files on our cohorts. Basically, data on the subjects in our master files matched the data from the municipal files on the basic health examination in terms of subject sex, name, and birthday. Data were available for 17,923 (33%) (6,532 (24%) men and 11,391 (42%) women) out of 54,498 subjects in Cohort I and 19,561 (31%) (6,598 (22%) men and 12,963 (41%) women) out of 62,398 subjects in Cohort II (Figure 1, (b+c)/(a+b+c+d)).

In this study, respondents (Figure 1, c and d) were divided into two groups by the basic health examination data: those for whom health examination data were available were defined as participants (Figure 1, c); those for whom the data were not available were defined as non-participants (Figure 1, d).

### Study variables

To describe characteristics of participants and non-participants in the basic health examination, we compared them on the basis of well-known or commonly used factors related to diseases^17^ including their sociodemographic factors (age, PHC area, educational background, and occupation), personal medical history (past history of diabetes mellitus, cerebrovascular disease, hypertension, ischemic heart disease, or cancer, family history of diabetes mellitus, cerebrovascular disease, ischemic heart disease (Cohort I), heart disease (Cohort II), or cancer, medication, and body mass index (BMI)) and lifestyle-related factors (vitamin supplement use, smoking status, alcohol drinking, sports and/or physical exercise in leisure time, and dietary habits).

Educational background was available for only Cohort I, and was grouped into three categories. The Japanese standard occupational classification was used to categorize occupation by open-ended question in Cohort I. In Cohort II, nine self-reported occupations were combined into the following 6 groups: employee and professional; agricultural, forestry, and fishery; self-employed; homemaker; unemployed; and other occupations. The subjects with 2 or more occupations across those groups were classified as a combination group. BMI was calculated from self-reported height and weight.^11^ Smoking status was classified as current, past, or never smokers, and pack-year was calculated.^12^ We defined non-drinkers (<1 day/month), occasional drinkers (1-3 days/month), and regular drinkers (1-2 days/week or more) based on the frequency of consumption in Cohort I. Non-drinkers were then subdivided into never-drinkers and ex-drinkers in Cohort II. Among regular drinkers, weekly ethanol consumption was calculated by combining the frequency per week and the usual daily amount of alcoholic beverages.^9^^,^^18^ The weekly intake frequency of 27 (Cohort I) and 33 (Cohort II) food items was reported in four (Cohort I) and five (Cohort II) categories.

### Statistical analysis

We excluded 29 subjects (9 in Cohort I and 20 in Cohort II) not of Japanese nationality (7 in Cohort I and 11 in Cohort II), or who had already moved away at the baseline, which we confirmed during the follow-up period (2 in Cohort I and 9 in Cohort II). Subjects aged 60 years and older in Cohort II were excluded because we matched age groups between the two cohorts. Finally, data for analyses included 43,140 subjects (20,658 men and 22,482 women) in Cohort I and 34,892 subjects (16,882 men and 18,010 women) in Cohort II. All analyses were performed separately according to sex and cohort.

Odds ratios (OR) and their 95% confidence intervals (CI) were calculated by unconditional logistic regression to estimate associations between participation in the basic health examination and sociodemographic factors, personal medical history, and lifestyle-related factors. Logistic regression analysis by SAS LOGISTIC procedure (SAS Institute Inc., Cary, NC) was used to adjust for age (40-44, 45-49, 50-54, and 55-59 years) and area (city vs. town and village) because the proportion of participants in the basic health examination varied between city, town and village.^19^ Multivariate ORs adjusted for age, educational background (only for Cohort I), and occupation were calculated. Participation in the basic health examination is influenced by occupation because the basic health examination in Japan does not cover subjects who have the periodical health examination (*Teiki-kenkou-shindan*) at their workplaces under the Industrial Safety and Health Law (*Roudou-anzen-esisei-hou*). To test the linear trend, consecutive integers were given to each category. All p values are reported as two-sided, and the significance level was set at p<0.05.

## RESULTS

Tables 1 and 2 show age- and area-adjusted ORs for participation in the basic health examination according to sociodemographic factors, personal medical history, and lifestyle-related factors in Cohorts I and II. In Cohort I, 5,883 (28%) men and 10,247 (46%) women underwent the basic health examination, against 2,787 (17%) men and 6,708 (37%) women in Cohort II.

**Table 1.**
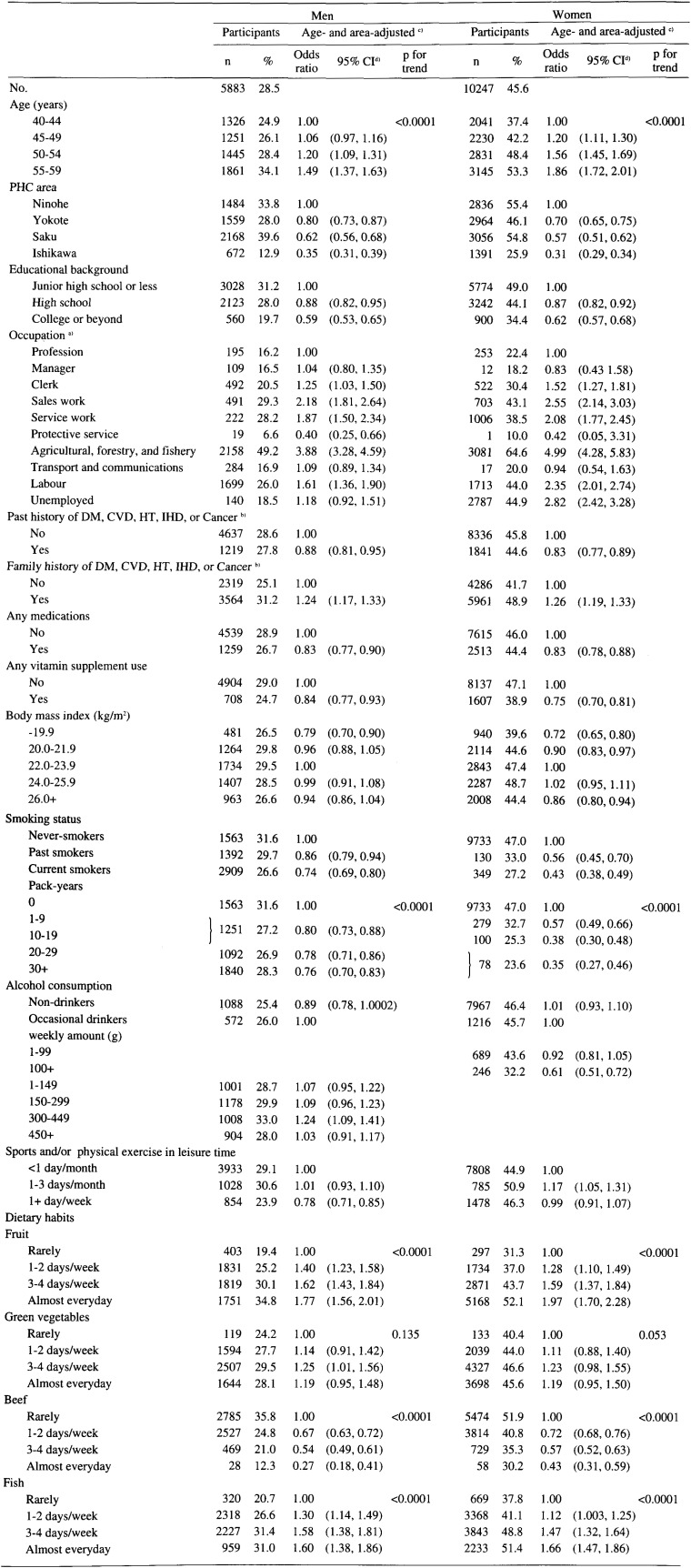
Proportions of participants and age- and area-adjusted odds ratios for participation in the basic health examination according to sociodemographic factors, personal medical history, and lifestyle-related factors in 1990: JPHC Cohort I.

a) The Japanese standard occupational classification was used to categorize occupation: professional and technical workers (Profession), managers and officials (Manager), clerical and related workers (Clerk), sales workers (Sales work), service workers (Service work), protective service workers (Protective service), agricultural, forestry, and fisheries workers (Agriculture, forestry, and fishery), workers in transport and communications occupations (Transport and communications), craftsmen, mining, production process and construction workers and laborers (Labor).

b) DM, diabetes mellitus; CVD, cerebrovascular disease; HT, hypertension; IHD, ischemic heart disease.

c) Adjusted for age in 1990 (40-44, 45-49, 50-54, 55-59) and area (city, town and village).

d) 95% CI: 95% confidence interval

**Table 2.**
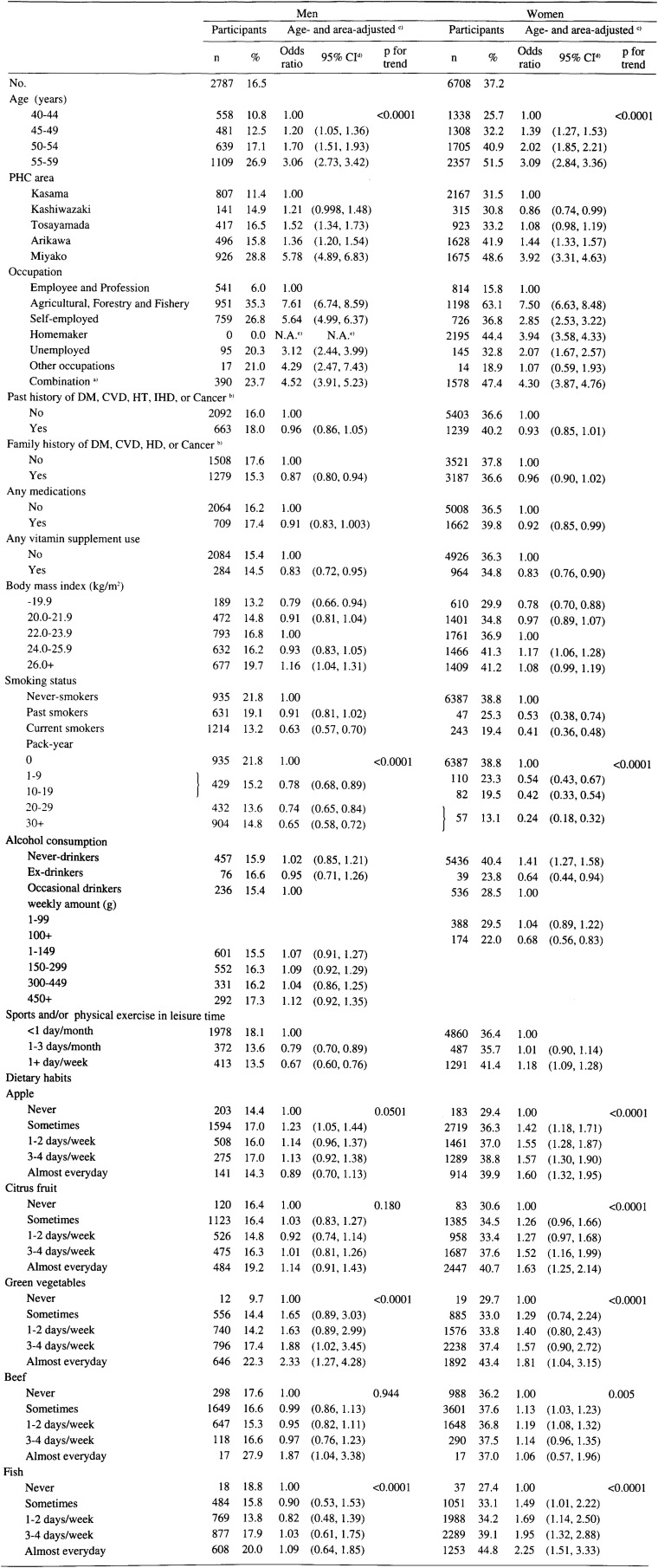
Proportions of participants and age- and area-adjusted odds ratios for participation in the basic health examination according to sociodemographic factors, personal medical history, and lifestyle-related factors in 1993-94: JPHC Cohort II.

a) Subjects with multiple occupations

b) DM, diabetes mellitus; CVD, cerebrovascular disease; HT, hypertension; IHD, ischemic heart disease; HD, heart disease.

c) Adjusted for age in 1993 (40-44, 45-49, 50-54, 55-59) and area (city, town and village).

d) 95% CI: 95% confidence interval

e) N.A., not available

### Sociodemographic factors

As commonly observed, participation in the basic health examination was associated with the age class, and the ORs for participation showed a significant increase with aging. In Cohort I, subjects categorized in higher educational background groups demonstrated statistically significant decreased ORs for participation in the basic health examination among both men and women. In Cohort I, subjects classified in Table 1 in the Clerk, Sales work, Service work, Agricultural, forestry, and fishery, or Labor categories had significantly higher ORs for participation in the basic health examination than those classified in the Profession category. In Cohort II, a significant elevation of ORs for participation in the basic health examination was observed for agricultural, forestry and fishery, self-employed, homemakers and unemployed groups compared to the employee and profession group.

### Personal medical history

Subjects with a past medical history had significantly lower ORs for participation in the basic health examination than those without such a history in Cohort I. A family history was associated with significantly increased ORs for participation in the basic health examination in Cohort I. However, an inverse association among men and no association among women were observed in Cohort II. The uses of any medication were related to the decreased ORs for participation in the basic health examination. Subjects of both sexes and cohorts in the lowest BMI category characteristically showed a significant decrease in ORs for participation in the basic health examination compared to subjects in the middle BMI categories.

### Lifestyle-related factors

The uses of any vitamin supplements were significantly related to the decreased ORs for participation in the basic health examination. Smoking status was related to significantly decreased ORs for participation in the basic health examination. As pack-year increased, ORs for participation gradually decreased regardless of sex or cohort and all p for trends reached significant levels. The combined variable of drinking habit and consumption was not related to participation in the basic health examination for men, especially in Cohort II. Female heavy alcohol drinkers (100+ g/week) demonstrated significantly lower ORs than occasional drinkers. In Cohort II, female never-drinkers had significantly higher ORs than occasional drinkers, whereas ex-drinkers had significantly lower ORs than them. Participation in the basic health examination was related to sports and physical exercise in leisure time, however, a different pattern was seen in both sexes. Statistically significant elevation of ORs for participation in the basic health examination was associated with increased frequency of fruit or apple and citrus fruit consumption with the exception of the men in Cohort II. A higher frequency of green vegetable intake was associated with significantly increased ORs in Cohort II, and a non-significant increase was observed in Cohort I. There were significantly decreased ORs related to increased frequency of beef intake in Cohort I. Statistically significant positive associations were observed between the frequency of fish intake and participation in the basic health examination (p for trend<0.0001), although clear association was not seen among men in Cohort II with respect to ORs in each category.

### Findings from multivariate analyses

Most personal medical history and lifestyle-related factors were almost unchanged even after adjustment for age, educational background (only in Cohort I) and occupation (data not shown). However, the statistically significant association disappeared among men for the following variables: past history (Cohort I), physical exercise in leisure time and fish (p for trend) (Cohort II). In addition, male drinkers (1-449 g/week) in Cohort I showed significantly elevated ORs compared to occasional drinkers: 1.22 (95%CI 1.07-1.38) for those drinking 1-149 weekly amount (g); 1.21 (95%CI 1.07-1.37) for those drinking 150-299 weekly amount (g); and 1.32 (95%CI 1.16-1.50) for those drinking 300-449 weekly amount (g). Among men in Cohort II, significantly increased ORs for participation in the basic health examination were observed for ‘Apple’ as follows: 1.32 (95%CI 1.11-1.56) for sometimes; 1.32 (95%CI 1.10-1.60) for 1-2 days/week; and 1.32 (95%CI 1.07-1.63) for 3-4 days/week. As for physical exercise in leisure time, the opposite finding of age- and area-adjustment was obtained among men in Cohort I (1.25 (95%CI 1.14-1.37) for 1-3 days/month and 0.95 (95%CI 0.87-1.04) for 1+ day/week). Significantly increased ORs were observed among women: in Cohort I, the corresponding ORs were 1.34 (95%CI 1.19-1.49) and 1.11 (95%CI 1.03-1.21); in Cohort II, the corresponding ORs were 1.14 (95%CI 1.01-1.30) and 1.30 (95%CI 1.19-1.41).

## DISCUSSION

In this study, the differences observed in some sociodemographic factors, personal medical history, and lifestyle-related factors between participants and non-participants might cause a selection bias. Criqui et al.^3^ pointed out that two types of bias can occur with substantial non-response in population-based prospective studies: (1) bias in the prevalence of exposure factors or disease due to the differential participation of individuals with given exposures or diseases, or (2) bias in risk rate ratio estimates determined by comparing the incidence rates of disease in the exposed and unexposed group. Our findings implied selection bias would lead to systematic error in estimating the prevalence of risk factors and diseases, thus limiting application of the results to participants in the basic health examination. In addition, estimation of exposure-disease associations might be affected in some cases. However, the degree of bias considered to be important depends on the intended use of the study results. As Greenland^20^ pointed out, “A bias that may have been considered insignificant in one context (such as in research establishing the existence of an association) may be considered more significant in a later context (such as in allocating of health care resources based on the strength of the association).”

Although inconsistent findings that might be due to the multiplicity of the comparison or the use of a different questionnaire between two cohorts were observed for several variables, our study basically showed that especially female participants had a favorable lifestyle profile, and obvious associations were observed for smoking and dietary habits. Similar results were reported in studies that targeted not only subjects for cancer screening programs^21^^-^^23^ but also the general population or some specific groups.^3^^-^^8^ With respect to studies on the basic health examination, Ozasa^24^^,^^25^ reported that participants had a healthier behavior, including better dietary habits such as limited salt intake, and eating more fish, vegetables, beans, fruit and seaweed. Some earlier studies^6^^,^^7^ showed that respondents had higher education and income than non-respondents. Meanwhile, we found that participants in the basic health examination tended to be older and less educated. They were more likely to engage in agriculture, forestry and fisheries or to be self-employed persons or homemakers in our study. This is partly because the basic health examination is provided free or at low cost by local municipalities to all people in Japan duly registered as residents who are 40 years old or more, and are not offered periodical health examinations at their workplace. Therefore, most retired, self-employed, or unemployed (including homemakers) persons underwent the basic health examination. In the present study, non-participants fell into two groups: those eligible to participate in the basic health examination who did not, and those who underwent the periodical health examination at their workplaces and thus had no need to take part in the basic health examination. However, in light of our study aim, we did not need to distinguish between two groups among non-respondents. Although the proportion of non-participants who underwent any health check-ups for the last year were 79% (79% in men and women) in Cohort I and 73% (76% in men and 69% in women) in Cohort II based on the baseline questionnaire, these conditions would lessen the associations between background characteristics and participation in the basic health examination. Nevertheless, relatively great differences were observed for smoking and dietary habits.

Regarding methodological issues, the strengths of the present study include the population-based large sample with high response rate obtained from nine PHCs that covered 27 administrative districts (city, town or village), although study areas were not selected randomly. Our results were principally consistent between the two cohorts. Thus, the external validity of our results might be assured for middle-aged Japanese population except for those living in urban areas. This indicates that attention must be given to interpretation and generalization of results from health examination participants in a rural community. To our knowledge, this is the first study to compare sociodemographic factors, personal medical history, and lifestyle-related factors between participants and non-participants in the basic health examination based on a large population-based sample in Japan.

In spite of these strengths, this study has several limitations. First, the differences in the respective questionnaires made it difficult to confirm consistent findings between Cohort I and Cohort II. This might in part explain the differences in findings between the two cohorts. Second, the subjects surveyed in this study were restricted to respondents to the baseline questionnaire. As mentioned above, non-respondents had an unhealthier lifestyle than respondents,^3^^,^^5^^,^^6^^,^^8^ and were less likely to participate in the basic health examination. In the present study, the proportions of non-respondents were 21% (24% in men and 18% in women) in Cohort I and 18% (21% in men and 14% in women) in Cohort II. In addition, participants (Figure 1, b) accounted for 16% (10% in men and 23% in women) of non-respondents in Cohort I and 7% (4% in men and 12% in women) in Cohort II (Figure 1, b/(a+b)). Although it could lead to underestimation of the association between participation to the basic health examination and sociodemographic factors, personal medical history, and lifestyle-related factors, this effect might be relatively small in this study, since nearly 80% or more of our subjects responded to the baseline questionnaire.

Despite these limitations, we found that female participants in particular had a different socioeconomic status from non-participants and showed a healthy lifestyle. These differences between participants and non-participants in the basic health examination might cause a selection bias that limits the application of the results to only participants in the basic health examination.

## APPENDIX

In addition to the initially mentioned investigators and their associated institutions in the JPHC Study Group (principal investigator: S. Tsugane), the study group members include the following: J. Ogata, S. Baba, T. Mannami, National Cardiovascular Center, Suita; K. Miyakawa, F. Saito, A. Koizumi, Y. Sano, Iwate Prefectural Ninohe Public Health Center, Ninohe; Y. Miyajima, N. Suzuki, S. Nagasawa, Y. Furusugi, Akita Prefectural Yokote Public Health Center, Yokote; H. Sanada, Y. Hatayama, F. Kobayashi, H. Uchino, Y. Shirai, T. Kondo, R. Sasaki, Y. Watanabe, Nagano Prefectural Saku Public Health Center, Saku; Y. Kishimoto, E. Tanaka, M. Kinjo, T. Fukuyama, Okinawa Prefectural Ishikawa (Chubu) Public Health Center, Ishikawa; K. Imoto, H. Yazawa, T. Seo, A. Seiko, F. Ito, Katsushika Public Health Center, Tokyo; A. Murata, K. Minato, K. Motegi, T. Fujieda, Ibaraki Prefectural Kasama (Mito) Public Health Center, Mito; K. Matsui, T. Abe, Niigata Prefectural Kashiwazaki Public Health Center, Kashiwazaki; M. Doi, Y. Ishikawa, A. Terao, Kochi Prefectural Tosayamada (Chuo-higashi) Public Health Center, Tosayamada; H. Sueta, H. Doi, M. Urata, Nagasaki Prefectural Arikawa (Kamigoto) Public Health Center, Arikawa; H. Sakiyama, N. Onga, H. Takaesu, Okinawa Prefectural Miyako Public Health Center, Hirara; F. Horii, I. Asano, H. Yamaguchi, K. Aoki, S. Maruyama, Osaka Prefectural Suita Public Health Center, Suita; S. Matsushima, S. Natsukawa, Saku General Hospital, Usuda; S. Watanabe, M. Akabane, Tokyo University of Agriculture, Tokyo; M. Konishi, Ehime University, Matsuyama; H. Iso, Y. Honda, Tsukuba University, Tsukuba; H. Sugimura, Hamamatsu University, Hamamatsu; Y. Tsubono, Tohoku University, Sendai; N. Kabuto, National Institute for Environmental Studies, Tsukuba; S. Tominaga, Aichi Cancer Center Research Institute, Nagoya; M. Iida, W. Ajiki, Osaka Medical Center for Cancer and Cardiovascular Disease, Osaka; S. Sato, Osaka Medical Center for Health Science and Promotion, Osaka; N. Yasuda, Kochi Medical School Nankoku; S. Kono, Kyushu University, Fukuoka; K. Suzuki, Research Institute for Brain and Blood Vessels Akita, Akita; Y. Takashima, Kyorin University, Mitaka; E. Maruyama, Kobe University, Kobe; and the late M. Yamaguchi, Y. Matsumura, S. Sasaki, National Institute of Health and Nutrition, Tokyo.
